# Scaphoid fracture: Bone marrow edema detected with dual-energy CT virtual non-calcium images and confirmed with MRI

**DOI:** 10.1007/s00256-017-2730-6

**Published:** 2017-07-29

**Authors:** Nazeer M. Dareez, Kristine H. Dahlslett, Eirin Engesland, Elisabeth S. Lindland

**Affiliations:** 1 Department of Radiology, SSHF Arendal, Postboks 783 Stoa, 4809 Arendal, Norway; 2 Department of radiology, Haukelands universitetssjukehus, Postboks 1400, 5021 Bergen, Norway

**Keywords:** Diagnosis of scaphoid fracture, Bone marrow edema, Dual-energy CT virtual non-calcium images, CT and MRI in occult scaphoid fracture, DECT VNCa images, Occult hip fracture, Vertebral compression fracture

## Abstract

We aimed to determine whether bone marrow edema (BME) in acute traumatic scaphoid fracture could be demonstrated with dual-energy CT (DECT) using MRI as the gold standard. In recent years, virtual non-calcium (VNCa) images have been used to demonstrate BME in trauma cases, for example, in vertebral compression fractures, hip trauma to detect occult fractures and knee fractures. We present three cases of acute scaphoid trauma. Two patients had subtle or invisible fractures on x-ray and conventional CT images, while DECT VNCa images clearly visualized the BME, which was confirmed by MRI. One patient had negative findings on both VNCa and MRI images. The DECT VNCa algorithm is a promising technique to demonstrate BME in scaphoid fractures, with potential for increasing the diagnostic value of CT in this type of injury.

## Introduction

Diagnosis of scaphoid fracture is often based on the history, clinical findings and radiography. However, immediately after injury, up to 25% of scaphoid fractures remain radiographically occult [1]. Displacement of more than 1 mm is considered a surgical indication [2], as there is a four-fold increased risk of non-union if these patients are immobilized in a cast compared with non-displaced immobilized fractures [3]. Concerns about complications such as non-union or osteonecrosis related to scaphoid fracture have led to the common practice of early immobilization and control with radiography within 7–10 days.

A meta-analysis by Carpenter et al. compared pooled sensitivity, specificity, and positive and negative likelihood ratios of clinical tests and different imaging modalities [1]. They found clinical tests such as snuffbox tenderness to have a good sensitivity (95%), but low specificity (39%). Follow-up radiography showed good specificity (93%), but low sensitivity (41%). In a systematic review, Malle et al. concluded that CT had almost equally high specificity for detection of scaphoid fractures (99%) compared with MRI (100%), but the sensitivity was lower than for MRI (72% versus 88%) [4]. Cost issues and accessibility limit the use of MRI.

Advances in DECT have improved the ability of CT to characterize and differentiate various body tissues. Substances with high atomic numbers such as iron, calcium and iodine show different attenuations at different energy levels of the x-ray spectrum, thus allowing for three-material decomposition, i.e. bone, fat and soft tissue [5]. This means that DECT has the ability to visualize tissue abnormalities in bone trauma such as microfractures, bleeding and edema, which represent bone bruise seen on imaging, also referred to as bone marrow edema (BME). Among the limitations of DECT is the spatial averaging effect. Marrow adjacent to the cortex may be filtered by software and edema may be masked; therefore, subcortical zones cannot be assessed by VNCa images [6, 7].

The technique has been used with encouraging results in other parts of the body.

We present the first case series reporting the use of DECT-VNCa images in acute scaphoid fracture.

## Materials and methods

**Fig. 1:**
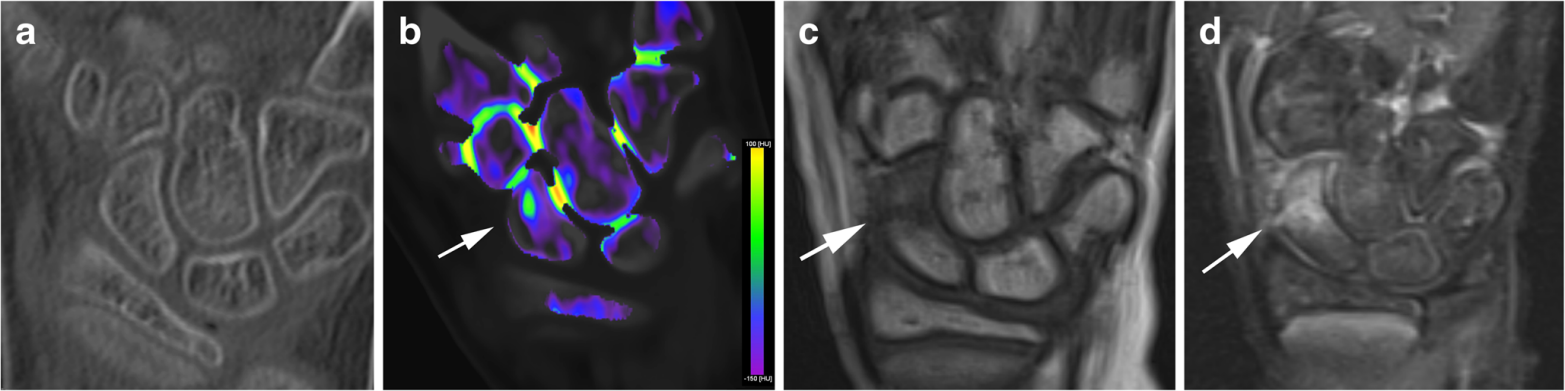
A 10-year-old female with non-displaced scaphoid fracture. CT coronal image with 2-mm slice thickness shows no fracture line (Fig. 1a). Coronal VNCa image demonstrates increased attenuation with a green color code relative to the more blue and purple normal bone, representing BME (Fig. 1b). On MRI images, the coronal T1-weighted sequence shows the low signal intensity area in the scaphoid waist (Fig. 1c) and corresponding high signal intensity on the coronal fluid-sensitive sequence (Fig. 1d), confirming the presence of BME

We present three patients who were referred to imaging with suspicion of scaphoid fracture. Written informed consent was obtained from these patients/their legal guardians for presenting the identifying information included here. In two of them, DECT-VNCa images clearly demonstrated BME, which was confirmed with MRI. All patients had a negative x-ray examination. A CT scan was performed with a dual-energy CT scanner (Somatom Definition FLASH, Siemens Healthcare, Forcheim, Germany) with 100- and 140-kV tube voltage, a tin filter, detector configuration 32 × 0.6 mm, pitch 0.5, rotation time 0.5 s, automated tube current modulation and fast scan mode. DECT reconstruction was done with a bone deconvolution kernel (B70f) and slice width/increment 1/0.7 mm. The DECT data set was post-processed and viewed in three planes with the SyngoVia software solution, Siemens Healthcare, Forcheim, Germany, with default parameters. The basis for the resulting images is the measurement of tissue attenuation at two different energy levels, the trabecular bone can be subtracted, and variations in bone marrow composition can be studied. The resulting CT number is color coded with the shift from blue/purple color representing fat to green color representing water and further to yellow color for increasing red marrow/blood contents, corresponding to an increasing CT number (the scale is included in Fig. 1b). This provides a visual assessment of the relative water content (green color) perceived as BME. The software gives an option to overlay VNCa images with standard CT images. We did not perform measurements of CT number.

All patients underwent an MRI scan with a 1.5-T system (Aera, Siemens Healthcare, Erlangen, Germany) with a flex coil and adapted protocol [8]: coronal T1 (TR 595 ms, TE 7.7 ms, FoV 140 mm, matrix 288 × 384, BW 250r, 3 mm slice thickness) and fluid-sensitive T1 tirm (TR 3500 ms, TE 23 ms, FoV 140 mm, matrix 192 × 256, 3 mm slice thickness).

## Case 1

A 10-year-old female was referred after a fall on an outstretched, dorsiflexed hand. She had pain at the first and second metacarpals and snuffbox tenderness. X-ray examination shortly after trauma was negative. During follow-up consultation after 6 days, there was still suspicion of a scaphoid fracture. A DECT scan was performed. Conventional CT (Fig. 1a) did not show any fracture, but there was a finding of BME in the scaphoid bone on VNCa images (Fig. 1b). This was confirmed 2 days later with MRI (Fig. 1c, d) as linear low signal intensity on the T1-weighted sequence and high signal intensity on fluid-sensitive images, consistent with a non-displaced fracture.

## Case 2

**Fig. 2:**
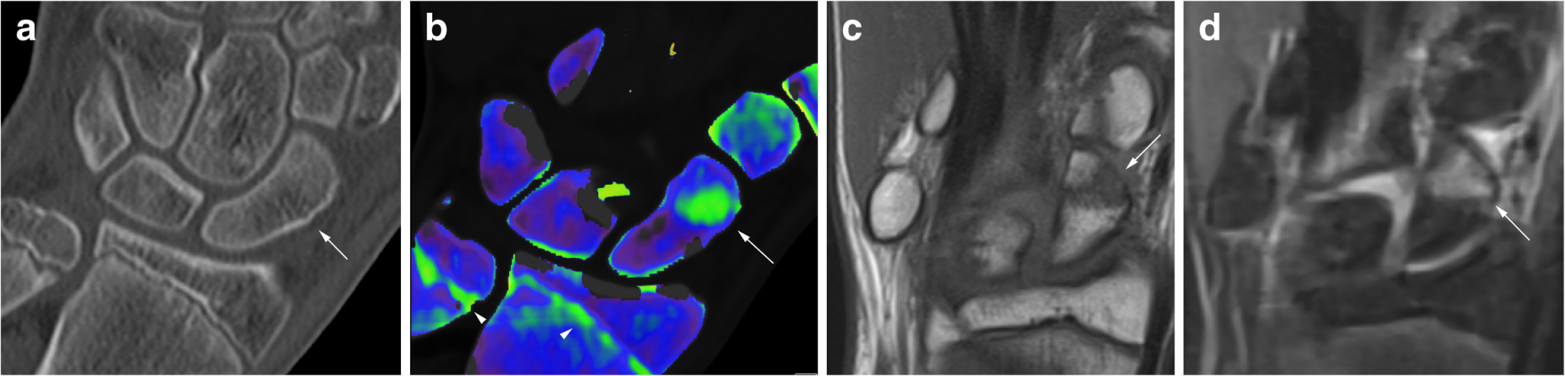
A 15-year-old male with a non-displaced scaphoid fracture shown as a subtle line on CT coronal reconstruction with 2 mm slice thickness (Fig.2a) and findings consistent with BME on the coronal VNCa image (Fig. 2b), color coded in green (arrow). Note the epiphyseal line on the distal radius, and the ulna is also color coded in green (arrowhead). MRI with coronal T1-weighted image (Fig. 2c) and coronal fluid-sensitive sequence (Fig. 2d) confirm BME

A 15-year-old male presented with snuffbox pain after falling on a dorsiflexed wrist during a handball match. An X-ray shortly after the injury at another hospital had been negative. After 2 days, he had swollen radial fingers and snuffbox tenderness and was referred for further imaging. At CT (Fig. 2a), there was a very subtle linear discontinuity in the scaphoid cortex, but no displacement. VNCa images (Fig. 2b) showed obvious BME. The finding was confirmed with MRI as low T1 signal intensity (Fig. 2c) and high signal intensity on the fluid-sensitive images (Fig. 2d), representing a non-displaced fracture.

**Fig. 3:**
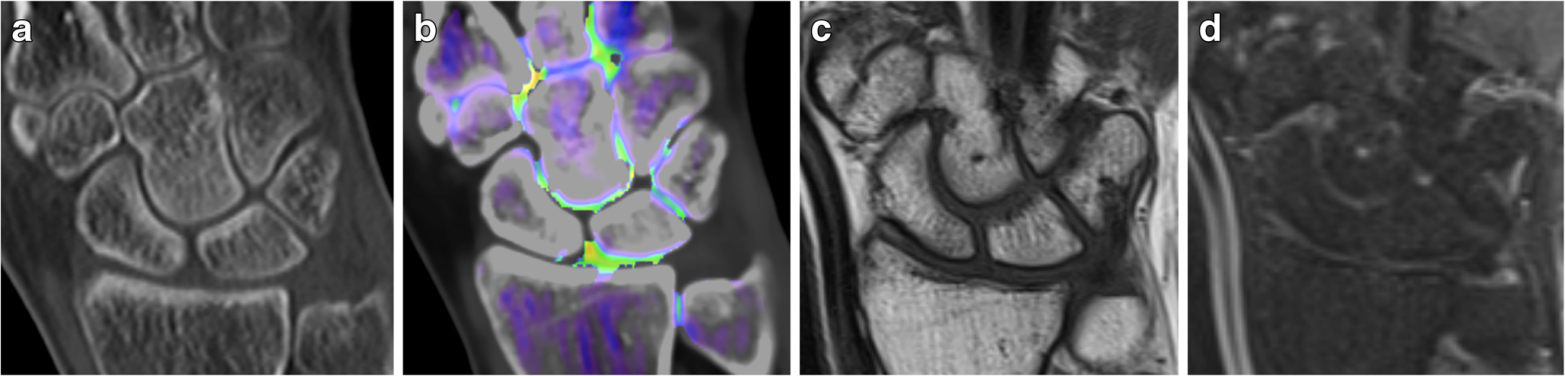
A 40-year-old female with a clinical suspicion of scaphoid fracture, but no fracture line seen on native CT (Fig. 3a) or any sign of BME on VNCa imaging (Fig. 3b) or MRI with coronal T1-weighted (Fig. 3c) and fluid-sensitive (Fig. 3d) sequences

## Case 3

A 40-year-old female carrying a sofa dropped it on her wrist. She was referred for imaging because of radial wrist pain. Clinical examination revealed distinct snuffbox tenderness, suspected to be scaphoid fracture. CT and MRI were performed the next day. There was no evidence of fracture on conventional CT and no BME on VNCa images. An MRI scan confirmed the negative findings (Fig. 3a-d).

## Discussion

Several studies of other anatomical regions have shown the utility of DECT VNCa in traumatic bone lesions. The technique is relatively novel. In 2010, Pache et al. demonstrated posttraumatic bone bruise of the knee with DECT VNCa images [5]. Their study included 21 patients and used MRI as the reference standard; the results for two readers were a sensitivity of 86.4% and specificity of 94.4%–95.5%. In a study of 14 patients with MRI and DECT-VNCa imaging, Ai et al. showed bone bruise persisting for up to 10 weeks after knee trauma [9]. Guggenberger et al. studied the diagnostic performance of DECT VNCa images for detecting traumatic bone marrow lesions of the ankle in 30 patients [10]. MRI was the reference standard, and they reported a high sensitivity of 90%, but a moderate specificity of 81.6%. Reddy et al. studied 25 patients with DECT VNCa for detection of occult, non-displaced hip fracture [11]. The reference standard was clinical follow-up at 30 days. The study showed a high sensitivity of 90%, but poor specificity of 40%. Five patients had no fracture, but three of them had positive findings in the VNCa images. Kellock et al. recently performed a retrospective study of 118 patients with suspected nondisplaced hip fracture. Three readers used VNCa images in addition to the standard CT bone reconstructions. They reported an up to 5% increase in sensitivity from 95% to 100% compared to standard CT alone, while specificity of 100% remained unchanged as it was already high with standard CT [6]. Reddy et al. attributed a low specificity in their study to degenerative changes of bone marrow in the femoral head or acetabulum, while Kellock et al. discussed whether this could be due to few patients without fracture. The technique has also been studied in vertebral compression fractures. Wang et al. studied 63 patients with 112 compression fractures using MRI as reference standard [7]. They found overall sensitivity of 63% and specificity of 98.5%. In a similar study of 23 patients with 72 vertebral fractures by Karaca et al., a sensitivity of 89.3% and specificity of 98.7% were reported [12]. Petritsch et al. recently studied 22 patients with 37 vertebral compression fractures [13]. They reported improved sensitivity from 64% to 92% when quantitative analysis of VNCa images by measuring CT numbers was used in addition to visual analysis. However, specificity with this quantitative method was 82.6% compared to 99.3% with qualitative visual assessment. In summary, the results for the anatomical regions studied so far are showing VNCa imaging to be a promising technique for the demonstration of bone marrow edema in skeletal trauma.

Choice of imaging method for the suspected scaphoid fracture depends on resource availability, local expertise, financial cost and how fast the diagnosis should be established, for instance, in athletes and workers who cannot tolerate occupational absence. Financial cost estimates should take into account not only the cost of imaging, but also the number of lost workdays and re-consultation.

Traditional practice with initial immobilization and clinical control with x-ray within 7–10 days means that three of four patients are immobilized unnecessarily, cf. 25% are radiographically occult. Current recommendations for a prompt diagnosis consider MRI to be the preferred imaging method [2]. A cost-analysis study has shown that an adapted protocol of MRI for scaphoid fracture is nearly equal to immobilization and repeat consultation [8].

The value of DECT-VNCa imaging primarily lies in its ability to help detect fractures, which may be subtle or undetectable on bone reconstruction CT images. Further studies are warranted to provide knowledge of the accuracy of CT combined with VNCa images for the diagnosis of acute scaphoid fracture. This can lead to progression toward more efficient patient care as well as less unnecessary loss of function and work absence after wrist trauma.

## Conclusion

The DECT VNCa imaging algorithm is a promising technique to demonstrate BME in acute carpal trauma. A short scanning time, no contraindication and availability in the emergency department setting provide wider clinical application than MRI. There is potential for increased sensitivity of CT in the diagnosis of acute scaphoid fracture when combined with such images. Demonstration of BME will facilitate the detection of non-displaced fractures and support subtle findings on conventional CT images.

## References

[1] Carpenter CR, Pines JM, Schuur JD, Muir M, Calfee RP, Raja AS (2014). Adult scaphoid fracture. Acad Emerg Med..

[2] de Weber K. Scaphoid fractures. UpToDate, Waltham, MA (Accessed 28 Feb 2017): UpToDate; Available from: https://www.uptodate.com/contents/scaphoid-fractures?source=search_result&search=scaphoid%20fracture&selectedTitle=1~25.

[3] Singh HP, Taub N, Dias JJ (2012). Management of displaced fractures of the waist of the scaphoid: meta-analyses of comparative studies. Injury..

[4] Mallee WH, Wang J, Poolman RW, et al. Computed tomography versus magnetic resonance imaging versus bone scintigraphy for clinically suspected scaphoid fractures in patients with negative plain radiographs. Cochrane Database Syst Rev. 2015 (6):Cd010023. 10.1002/14651858.CD010023.pub2 PMC6464799 26045406

[5] Pache G, Krauss B, Strohm P (2010). Dual-energy CT virtual noncalcium technique: detecting posttraumatic bone marrow lesions—feasibility study. Radiology..

[6] Kellock TT, Nicolaou S, Kim SS, et al. Detection of bone marrow edema in nondisplaced hip fractures: utility of a virtual unenhanced dual-energy CT application. Radiology. 2017:161063. 10.1148/radiol.2017174014 28825880

[7] Wang CK, Tsai JM, Chuang MT, Wang MT, Huang KY, Lin RM (2013). Bone marrow edema in vertebral compression fractures: detection with dual-energy CT. Radiology..

[8] Dorsay TA, Major NM, Helms CA (2001). Cost-effectiveness of immediate MR imaging versus traditional follow-up for revealing radiographically occult scaphoid fractures. AJR Am J Roentgenol..

[9] Ai S, Qu M, Glazebrook KN (2014). Use of dual-energy CT and virtual non-calcium techniques to evaluate post-traumatic bone bruises in knees in the subacute setting. Skeletal Radiol..

[10] Guggenberger R, Gnannt R, Hodler J (2012). Diagnostic performance of dual-energy CT for the detection of traumatic bone marrow lesions in the ankle: comparison with MR imaging. Radiology..

[11] Reddy T, McLaughlin PD, Mallinson PI (2015). Detection of occult, undisplaced hip fractures with a dual-energy CT algorithm targeted to detection of bone marrow edema. Emerg Radiol..

[12] Karaca L, Yuceler Z, Kantarci M (2016). The feasibility of dual-energy CT in differentiation of vertebral compression fractures. Br J Radiol..

[13] Petritsch B, Kosmala A, Weng AM (2017). Vertebral compression fractures: third-generation dual-energy CT for detection of bone marrow edema at visual and quantitative analyses. Radiology..

